# The Establishment of a precise intelligent evaluation system for sports events: Diving

**DOI:** 10.1016/j.heliyon.2023.e21361

**Published:** 2023-10-20

**Authors:** Ning Hao, Sihan Ruan, Yiheng Song, Jiashun Chen, Longgang Tian

**Affiliations:** a School of Civil Engineering, Southeast University, Nanjing, 211189, China; b School of Engineering, RMIT University, Melbourne, 3001, Australia; c School of Engineering, The University of Tokyo, Tokyo, 113-8654, Japan; d School of Computer Science and Engineering, Southeast University, Nanjing, 211189, China

**Keywords:** Action recognition and assessment, Computer vision, Engineering mechanics, Diving

## Abstract

The introduction of action quality assessment technology in sports events to achieve precise intelligent evaluation can greatly enhance the objectivity and effectiveness of competition results. Taking diving as the specific application background, this study proposes a novel Multi-granularity Extraction Approach for Temporal-spatial features in judge scoring prediction (MEAT) under the conditions of action quality assessment. On the one hand, it uses dual-modal inflated 3D ConvNet to extract the temporal and spatial features of each modal diving video at the video granularity parallelly and to merge them to form a global feature. On the other hand, the human body pose is modeled, and the simulated athlete's three-dimensional splash state is taken as local characteristics at the object granularity. Finally, the global and local features are concatenated into the fully connected layer, and heuristic method inspired by competition rules using labeled distribution learning are employed to output the probability distribution of the average score of all referees. The maximum probability score is selected and multiplied by the difficulty coefficient to obtain the final diving score. Through comprehensive experiments, comparing the Spearman's rank correlation (SRC) evaluation results of existing methods on the UNIV-Dive dataset, this framework reflects the greater accuracy advantage and further lays the foundation for the actual implementation of the technology.

## 1 Introduction

Action quality assessment (AQA) technology can be widely applied to many realistic scenarios, such as intelligent sports referees, surgical skill scoring, human pose estimation [[1], [2], [3]] and sports therapy guidance [4,5]. Especially in sports events, an accurate and intelligent evaluation and scoring system will greatly enhance the objectivity and effectiveness of the competition results and then promote the fairness of the competition. There have been related reports that the referee's scoring has been questioned [6,7]. Therefore, there is an increasing demand for the development of intelligent evaluation systems for sports events. This study aims to generate accurate and objective results based on live video intelligently so that athletes who have worked hard for decades for a project will not be treated unfairly.

For the intelligent evaluation system of sports events, especially diving, some scholars have carried out research and made some successful explorations [[8], [9], [10]]. However, existing algorithms are primarily based on deep learning. These methods overly depend on pose features in the video, while overlooking local visual quality clues crucial for scoring, such as the splashing conditions in diving. Although attention mechanisms and Transformers can extract long-range dependencies in videos, the effectiveness of these techniques is not significant due to the scale of the existing diving datasets. Moreover, these methods do not sufficiently utilize key parameters provided in the dataset, such as difficulty coefficients. The capture and evaluation accuracy of the overall action smoothness still needs improvement.

Therefore, to overcome the limitations of existing methods, this study proposes a novel Multi-granularity Extraction Approach for Temporal-spatial features in judge scoring prediction (MEAT). It selects the universal UNIV-Dive as the dataset [8], closely follows the rules of the Olympic diving competition, re-examines the video spatio-temporal modeling in AQA and divides it into three stages from the technical level. 1) Improve the existing technology based on the dual-modal inflated 3D ConvNet (I3D), extract the spatio-temporal features of diving videos in RGB and Flow modes, and fuse them to form global features. 2) Extract the pose when the athlete is about to enter the water (touch the water surface), use the extracted features to reconstruct the three-dimensional geometric model of the human body, import the geometric model into the preestablished finite element mechanical model based on the coupled Eulerian Lagrangian (CEL) algorithm to simulate the water surface change (three-dimensional splash shape) after the athletes enter the water, and finally obtain the splash feature as a local feature. 3) Integrate video and object granularity, concatenate global and local features, and pass them into the fully connected layer. Different from the previous methods, this study does not directly predict the diving score but predicts the probability distribution of the referee's average score (dividing the final diving score by the difficulty coefficient). The first stage makes full use of the existing I3D model and adds an optical flow on this basis, which can better capture the coherent attributes of the overall movement. In the second stage, the fluid‒solid coupling simulation method in mechanics is applied to the weak point of the neural network algorithm, and combined with dynamic-explicit analysis, an accurate and rapid solution to the splash state is realized, which is the innovation of this paper. The third stage is based on a heuristic method inspired by competition rules using label distribution learning (LDL), improving the output strategy of the fully connected layer and innovatively solving the diving score. The method has been patented [11].

The main contributions of our work are as follows.

1) Multi-granularity feature extraction. This research paper presents an avant-garde method for extracting features predicated on both video and object granularities. Employing dual-modal I3D, the approach extracts spatio-temporal features from the granularity of diving video sequences, while the local features at the object granularity level are derived from a simulated three-dimensional splash state, obtained via FEA modelling, as the diver enters the water. This innovative method of multi-granularity feature extraction renders a more comprehensive representation of the movements and states of a diver, thereby amplifying the model's predictive accuracy.
2) Novel judge scoring prediction model. This study introduces a heuristic method inspired by competition rules, based on LDL. Rather than directly forecasting diving scores, it generates the probability distribution of the average scores given by referees. Through this inventive approach, the model is capable of more accurately predicting actual scoring scenarios.
3) Outstanding experimental results. On the UNLV-Dive dataset, the performance of the proposed method outperforms all other contemporary methods. This exemplifies the distinctive advantages of this innovative framework in the domain of diving quality evaluation.

The limitation of our work is as follows:

The acquisition of the three-dimensional splash state is conducted through an offline process, which could lead to a slight reduction in processing efficiency.

## 2 Related work

### 2.1 Action quality assessment

At present, the vast majority of action quality assessment work uses methods based on deep learning [12,13], which extracts local or global features from video data, then uses fully connected layers or other feature fusion methods to aggregate these features. Ultimately, methods such as regression, pairwise ranking, or label distribution learning are used to generate assessments of video quality. For example, Pirsiavash et al. [9] were the first to apply deep learning to intelligent diving assessments, proposing the use of discrete fourier Transform/discrete cosine Transform (DFT/DCT) of body posture as features for support vector regressors (SVR), to map to the final action quality score. Subsequently, Venkataraman et al. [14] improved upon Pirsiavash et al.‘s work by using approximate entropy to better encode postures, thereby enhancing results. Recently, the Convolutional 3D (C3D) network [15] has shown excellent performance in action recognition tasks, owing to its ability to capture spatio-temporal features of appearance and significant actions. Parmar and Morris [8] believe this helps extract visual cues and proposed three frameworks - C3D-SVR, C3D-LSTM, and C3D-LSTM-SVR. The results they obtained were better than previous models, proving the effectiveness of C3D in intelligent diving assessment. Xiang et al. [16] proposed breaking down video clips into specific action segments and fusing average segment features, improving performance by adding more refined segment labels to the data samples. Li et al. [17] divided samples into 9 segments and used 9 different C3D networks to specifically extract features of different segments. These features were connected and further processed through convolutional kernels and fully connected layers, using ranking loss and L2 loss (Mean Squared Error) to generate final diving scores. Since C3D only uses single modality for feature extraction and does not consider information from other modalities of the video, many researchers currently use the dual-modal I3D as a backbone. For instance, Yu et al. [18] extracted video features using the I3D network in segments and then used a pairwise regression method to first learn the differences of the input video from the example video, and then added the difference scores to the example video score to get the action quality evaluation results. Similarly, Wang et al. [19] used the I3D network as a video representation framework and proposed a spatio-temporal pipeline method to obtain complex and varied action location features, finally predicting action scores through regression. However, these methods heavily rely on the posture features inherent in the video and neglect the local visual quality cues that are crucial to the final score, such as the splash of water in diving.

Recently, some studies have incorporated attention mechanisms and Transformers to extract long-range dependencies in videos. Parmar et al. [10] treated diving scoring as a multi-task regression problem and collected the largest multi-task AQA dataset to date, which includes 1412 diving samples. The multi-task labels include diving scores, action recognition, and summary generation. Wang et al. [20] employed spatial convolutional networks (SCN) and temporal convolutional networks (TCNs) with a two-stage training strategy. Specifically, they introduced attention mechanisms in the temporal convolution to fuse features based on the impact of temporal dimension features on overall performance. Zeng et al. [21] trained two graph convolutional network units and one attention unit, where the former represents dynamic information and pose correlations, and the latter assigns weights to each pose accordingly. Xu et al. [13] segmented the example and query videos and input the corresponding video clips into the same Transformer decoder, finally summing them to obtain the action quality assessment results. However, these methods have significant limitations because the effectiveness of attention mechanisms and Transformers relies on large volumes of data [22], whereas diving datasets usually have only a few thousand training samples. Attention mechanisms and Transformers themselves lack inductive bias, and their blind usage may have a negative impact on the model's ultimate performance. Therefore, we still adopt the currently popular I3D video feature extraction framework as our backbone. On the other hand, these methods have not fully utilized the objective parameters provided by the dataset, such as difficulty coefficients, and lack accuracy in capturing the overall smoothness of actions.

### 2.2 Label distribution learning

Label distribution learning (LDL) is a novel machine learning paradigm where the model's description of all labels forms a structure resembling a probability distribution. It focuses on learning the sample distribution rather than individual labels. It was initially proposed by Geng et al. [23] for facial age estimation using LDL. They introduced the improved iterative scaling - learning from label distribution (IIS-LLD) and conditional probability neural network (CPNN) algorithms to extract facial features. Later, with the development of deep learning, more and more methods rely on deep learning and Label Distribution Forests for label distribution learning. These methods have been widely used in tasks such as facial keypoint detection [[24], [25], [26], [27]], and crowd detection [28]. In the context of action quality assessment, Tang et al. [29] proposed using the LDL method to predict the score distribution of input action videos instead of a single score, to handle severe score uncertainty. However, this approach significantly limits the performance of AQA tasks. In terms of video distribution, Geng et al. [30] introduced a soft grammar parsing method for video parsing, describing video segments using different sub-actions. Ling et al. [28] used a mixture of Gaussian distributions to model the variation in crowd count in different video frames, for indoor crowd counting. In the field of AQA, Zhang et al. [31] proposed a novel pseudo-subscore learning (PSL) method based on label reconstruction. In this method, the total score of an action is not only used as the quality label but also as a feature for the training set. A label reconstruction-based learning algorithm was established to generate pseudo subscore labels for the training set. In this study, based on the innovative LDL-heuristic method inspired by competition rules, we convert diving scores into label distributions using Gaussian distribution functions. These label distributions are then used as supervised information in the loss function to constrain the predicted results. This allows for a more rational optimization of model parameters and provides a theoretical basis for providing more accurate action quality assessment results.

## 3 Method

### 3.1 Heuristic method inspired by competition rules

Diving referees usually have years of training to evaluate diving quality according to complex basic rules. If machines are used for AQA, similar rules must be followed. Therefore, it is necessary to be familiar with diving rules before developing an intelligent evaluation system.

In the diving competition, the referees evaluate the scores according to the athletes' approach (i.e., walking board and running platform), take-off, aerial movements and water entry movements. Delete the highest and lowest scores and multiply the sum of the remaining scores by the difficulty coefficient of the jumping movement of the athlete to obtain the actual score of the action [11]. This rule shows that each referee has different ruling preferences, and the scores given are different, but they are also consistent according to the level of the athlete's diving process. This property can naturally be regarded as a normal probability distribution, the center of which is the average of all referees' scores, which can be perfectly calculated by LDL.

In addition, observing the diving rules, it can be found that the difficulty coefficient is an objective value, which indicates the difficulty of the athlete's completion of the action. International diving competition rules determine the corresponding degree of difficulty for each diving action. It determines the value according to differences in action groups, competition events (springboard, platform), equipment height, action posture, and the number of cycles of somersaults. Inspired by this, it is only necessary to predict the average score of all referees and then multiply it by the action difficulty coefficient to predict the final diving score.

This study combines deep learning and simulation to evaluate the quality of diving, extracts global features from diving videos, extracts local features through finite element mechanics simulation, and combines the above two to maximize the real scoring effect. Using the UNLV-Dive dataset [8], there are 370 video materials, including the men's 10 m platform diving semifinals and finals of the 2012 Olympic Games. Each video contains two labels, namely, the difficulty coefficient *DD* and the diving score *S*. The diving videos are all shot from the same side view with little change in perspective. This dataset also splits the training and testing sets 300/70.

### 3.2 Global feature extraction at video granularity

**Fig. 1:**
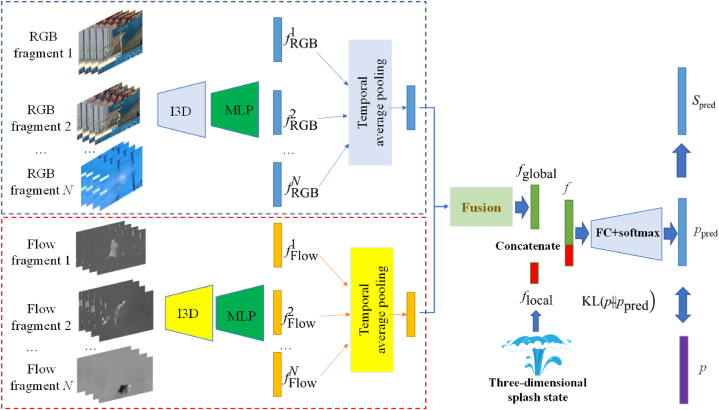
An intelligent evaluation process of diving quality. First, utilizes deep learning methods to extract global features from diving videos, then applies FEA to extract local features. Finally, by integrating these two types of features, a realistic scoring evaluation of the quality of diving is carried out.

As shown in Fig. 1, a diving video in RGB mode with *L* frames $V_{\text{RGB}}={\left\{F_{\mathrm{RGB}}^{l}\right\}}_{l=1}^{L}$ is given. Considering that the optical flow is the instantaneous speed of the pixel movement of the spatially moving object on the observation imaging plane, it can effectively represent the motion information of objects between adjacent frames. Therefore, we first use the classic TV-L1 optical flow algorithm to convert it into an optical flow mode $V_{\mathrm{Flow}}={\left\{F_{\mathrm{Flow}}^{l}\right\}}_{l=1}^{L}$. Then, the sliding window strategy is used to divide both *V*_RGB_ and *V*_Flow_ into *N* overlapping segments. The *V*_RGB_ and *V*_Flow_ segments are input into an I3D [32] model, and then a pair of RGB and Flow segment features $\left\{f_{\text{RGB}}^{1},f_{\text{RGB}}^{2},\cdots ,f_{\text{RGB}}^{V}\right\}$, $\left\{f_{\text{Flow}}^{1},f_{\text{Flow}}^{2},\cdots ,f_{\text{Flow}}^{V}\right\}$ are output through an MLP block composed of 3 fully connected layers. It should be noted that the weights of these 3 fully connected layers are shared by all segments. Then, for the extracted fragment features, use temporal average pooling to fuse them into *f*_RGB_ and *f*_Flow_, respectively, and further averagely mix them into a global feature *f*_global_.

### 3.3 Local feature extraction at object granularity

Key objects in diving, such as splashes and their shapes (i.e., height and radius), are an important basis for referees' decisions. If the water splash shape can be calculated in advance and used as a local feature of the video, it may improve the objectivity of the prediction score. The detailed process of obtaining the splash state is described below. Further, to enrich the dimensional information of the splash, we simulate the athlete's diving posture to obtain a three-dimensional splash. The detailed process of obtaining the splash state is elaborated on below.

#### 3.3.1 Extraction of key frames of water entry and creation of the human body's 3D pose

First, it is necessary to extract the frame where the athlete just entered the water. Once the frame is determined, it is convenient to extract the human body pose when entering the water and perform modeling and simulation. Target detection is performed on the human body, and the human body is integrated with the water after entering the water. In this case, the human body cannot be detected. Therefore, the yolov5 model is used to detect human objects in each frame of the video. If no human body is detected in consecutive frames after a certain frame, this frame is likely to be a key frame where a person just entered the water. Extract the key information of the human body pose through the key frame picture.

**Fig. 2:**
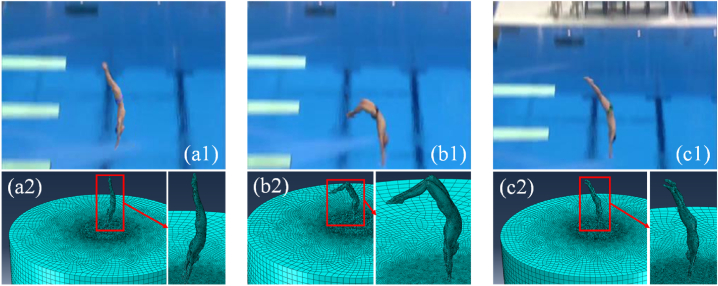
Videos and finite element analysis models.

After obtaining the human body pose through the key frame picture (Fig. 2a1, b1, c1), it is necessary to construct a 3D model of the human body (Fig. 2a2, b2, c2). In the actual application process, first, data are collected before the game, including the athlete's height, weight, and body shape. Combined with the key frame information of the video, it is modeled in the professional human body modeling software Daz Studio 4.12. The secondary development of the software can realize rapid parametric modeling to obtain the calculation model and initial data of the finite element simulation.

In this study, three diving videos in the aforementioned dataset UNLV-Dive are selected as examples. During the simulation process, the average body data of athletes are used for modeling, and the model is saved in. sat format, as shown in Fig. 2.

#### 3.3.2 Finite element mechanics simulation

After obtaining the sports data and body data of the athletes during the diving process, finite element simulation analysis is carried out. Through the simulation results, specific references are provided for scoring. In the actual application process of the project, the collected simulation data and the athlete's body model are automatically input into the analysis software through the program method. Under various preset parameter conditions, simulation calculations and output results are quickly performed. This study will introduce the parameter settings in the simulation process in detail.

**Fig. 3:**
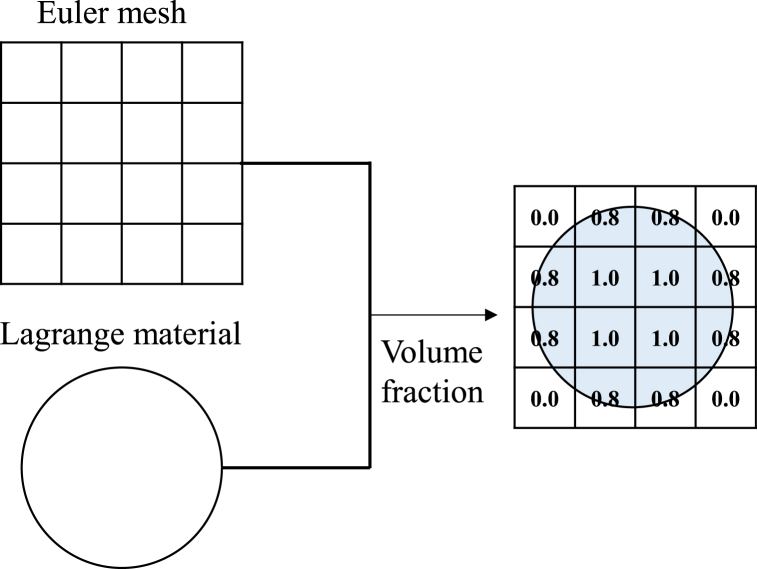
The fundamental principle of the CEL algorithm.

This study employs the CEL algorithm (integrated within the ABAQUS 2020 finite element analysis software) for simulation analysis. Its basic principle is shown in Fig. 3. The Eulerian description uses a fixed spatial grid, adapting to large deformation scenarios, while the Lagrangian description follows material movement, therefore, it is very suitable for describing the behavior and distribution of materials [33]. When the two are coupled, we can freely assign materials to the Eulerian grid based on volume fractions, providing accuracy when simulating complex interactions between liquids and solids. We use this method to simulate the three-dimensional splash shape when the athlete enters the water [34].

When creating analysis components, the human body model is imported and a sufficiently large water body and splash area are constructed to avoid the water body boundary interfering with the formation of the splash shape. Here, the human body is considered as a discrete rigid body (Lagrangian body), and the water body is treated as a Eulerian body to allow flow and deformation within Euler's fixed spatial grid. The material properties of the water part are shown in Table 1. This integrated use of Euler's grid and Lagrangian material characteristics enables us to more accurately simulate and predict the splash shape when the athlete dives into the water.

**Table 1:** Material properties of the water part.

Density/(kg·m^−3^)	Sound speed/(m·s^−1^)	Viscosity/(kg·m^−1^·s^−1^)
1000	1483	0.01

**Fig. 4:**
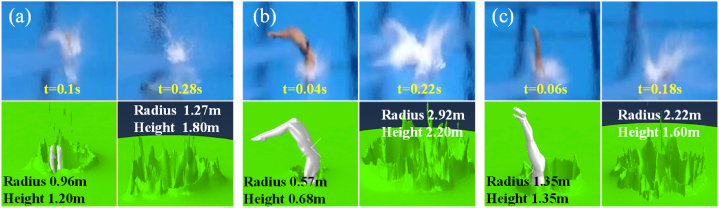
Splash shape comparison. (a) Video 1. (b) Video 2. (c) Video 3.

The materials are given to the components. When assembling the components, the athlete model is placed close to the water surface. By giving the initial velocity, the effect of the athlete's free fall is simulated. Using the dynamic-explicit solver, gravity is set, the boundary of the water part is constrained, and a predefined field is set in the default analysis step to distinguish the water part in the Eulerian body and the splash area. For dividing the mesh, when the mesh density of the core area is below 0.02, the water splash effect is obvious, the difference is not large, and the energy change is stable, so the mesh density of the core area can be 0.02. By simulating, we can obtain the splash shape of the three mannequins when they jump into the water and compare it with the splash shape in the material video, as shown in Fig. 4.

From the comparison of Fig. 4a–c, it is found that the splash height, radius and density of the three-dimensional splash produced by the simulation are similar to those in the video, but there are certain differences in the splash shape. A possible reason for the difference is the lack of accurate athlete model data, thus causing fluctuations in the final results. In the actual application process, it can be solved by collecting data before the competitions and finally improving the accuracy of the simulation. In conclusion, the final effect of the above simulation is closer to the actual effect in the video, and the simulation results meet the experimental requirements.

### 3.4 Diving score prediction

The global features obtained in Section 3.2 and the local features obtained in Section 3.3 are concatenated into a video comprehensive feature *f* through dimension splicing. This feature not only contains a wealth of global information about the athlete's motion sequence but also reflects the local features of the splash state after diving, which can well represent the diving video. Pass *f* to the fully connected layer. Unlike previous methods, this study does not directly predict the diving score, but rather employs a heuristic method inspired by competition rules based on LDL, for which the reason is provided in section 3.1. It predicts the probability distribution of the average score given by all referees (diving score divided by the difficulty coefficient). First, a score probability distribution is generated to represent the real distribution, and the prediction accuracy is continuously improved by optimizing the predicted probability distribution and the loss function of the real probability distribution. Finally, the difficulty coefficient is multiplied to predict the score.

#### 3.4.1 Prior probability distribution generation

In the training phase, given the diving video with the marking score *S* and the difficulty coefficient *DD*, the average score of the referee is *S*_J_=*S*/*DD*. As shown in Equation (1), a Gaussian function with mean *S*_J_ and standard deviation *σ* is first generated as a prior probability distribution.

(1) $$g\left(c\right)=\frac{1}{\sqrt{2\pi \sigma }}e^{\frac{{\left(c-S_{J}\right)}^{2}}{2\sigma^{2}}}$$

*σ* is a hyperparameter used to estimate the degree of uncertainty of *S*_J_. By discretizing the score range uniformly into score sets *c* = [*c*_1_, *c*_2_, …, *c*_m_], the degree coefficient of *S*_J_ on each score is represented by the vector *g* = [*g*(*c*_1_), *g*(*c*_2_), …, *g*(*c*_m_)]. The final *S*_J_ probability distribution *p* = [*p*(*c*_1_), *p*(*c*_2_), …, *p*(*c*_m_)] is realized by normalizing *g*, and the calculation of each element in *p* is reflected in Equation (2).

(2) $$p\left(c_{i}\right)=\frac{g\left(c_{i}\right)}{\sum_{j=1}^{m}g\left(c_{j}\right)}\;i=1,2,\ldots ,m$$

#### 3.4.2 Loss function

Input *f*_global_ to a fully connected layer and a softmax layer, and then map to the *m*-dimensional probability distribution *p*_pred_ = [*p*_pred_(*c*_1_), *p*_pred_(*c*_2_), …, *p*_pred_(*c*_m_)] as the predicted probability distribution. Finally, the loss function is obtained by calculating the KL divergence between *p*_pred_ and *p*, which is shown in Equation (3).

(3) $$\text{KL}\left(p\|p_{\text{pred}}\right)=\sum_{i=1}^{m}p\left(c_{i}\right)\log\frac{p\left(c_{i}\right)}{p_{\text{pred}}\left(c_{i}\right)}$$

#### 3.4.3 Diving score calculation

In *p*_pred_, the score with the largest probability value is selected as the average score *S*_J, pred_ of all referees (Equation (4)) and multiplies by the difficulty coefficient *DD* to obtain the final predicted diving score *S*_pred_, which is shown in Equation (5).

(4) $$S_{J,\text{pred}}={\mathrm{argmax}}_{c_{i}}\left\{p_{\text{pred}}\left(c_{1}\right),p_{\text{pred}}\left(c_{2}\right),\ldots ,p_{\text{pred}}\left(c_{m}\right)\right\}$$

(5) $$S_{\text{pred}}=DD\times S_{J,\text{pred}}$$

## 4 Experiment

### 4.1 Evaluation index

Action quality evaluation is used as a regression problem to predict quality “score”, so this study uses Spearman's rank correlation (SRC) [[8], [9], [10],^17^,^20^,^29^] used in previous literature as the evaluation index. The range of SRC is between −1 and 1. The higher the SRC, the stronger the correlation between the real score and the predicted score. That is, the closer the SRC is to 1, the stronger the correlation, and vice versa.

### 4.2 Implementation details

The Pytorch deep learning framework is used to write the model on the Ubuntu 16.04 system, and iterative training is performed 100 times (through an Nvidia TiTian RTX GPU to accelerate the training). Using the pretrained I3D model (including RGB and Flow modes) on Kinetics as a video feature extractor, it takes a 16-frame action sequence as input and outputs 1024-dimensional features. The yolov5 model, pre-trained on the COCO dataset, is utilized to detect the key frames of entering the water. Fill each UNIV-Dive dataset to 151 frames (videos with insufficient frames are supplemented with all zero frames). Each video is divided into 10 segments using a sliding window strategy. Each MLP block contains two hidden fully connected layers FC (256, ReLU) and FC (128, ReLU). The network is optimized with the Adam optimizer, and the learning rate is set to 0.0001. In addition, in the experiment, this study normalizes the final total score to the range of [0, 50] to ensure scale consistency and applies a Gaussian distribution to obtain the prior probability distribution.

### 4.3 Experimental results

Comparing the performance of our method with several other state-of-the-art methods, Table 2 shows the results evaluated with SRC. It can be seen that on the UNLV-Dive dataset, the methods in this study are superior to other existing methods. This result demonstrates the unique advantages of our innovative framework in diving quality assessment.

**Table 2:** Performance of different methods on UNLV-Dive.

Method	SRC	Published year
C3D + LSTM [8]	0.2700	2017
Pose + DCT [9]	0.5300	2014
C3D + SVR [8]	0.7800	2017
C3D + CNN [17]	0.8009	2018
USDL [22]	0.8147	2020
SCN + ATCN [20]	0.8500	2020
S3D [16]	0.8600	2018
C3D-AVG-MTL [10]	0.8808	2019
TSA-Net [19]	0.8832	2022
ESL + FTPS [31]	0.8700	2023
Ours (MEAT)	0.9072	

### 4.4 Ablation study of each module in this method

#### 4.4.1 Global ablation

To further analyze the effectiveness of each module in the model proposed in this study, we carried out ablation experiments on three main steps, i.e., global features, local features, and the LDL module. The symbol **√** denotes that the overall features incorporate global/local features, or the LDL method is employed in the prediction process. Conversely, the symbol **×** implies that global/local features are not incorporated, or that the prediction method relies on conventional regression for score prediction as cited in Ref. [9]. The results of the ablation experiment show that even after each module is individually removed, the method can still achieve comparable results for action quality evaluation with previous methods (Table 3). However, compared with the complete method, the SRC decreases to a certain extent after each module is removed, which fully demonstrates the effectiveness and importance of each module. The degree of decrease is ranked from high to low as global features, local features, and LDL.

**Table 3:** Ablation experiment results of the three main steps of this method on the UNLV-Dive dataset.

Method	Global Features	Local Features	LDL	SRC
1	×	√	√	0.7211
2	√	×	√	0.8725
3	√	√	×	0.8846
4 (Complete)	√	√	√	0.9072

#### 4.4.2 Effectiveness of dual-path I3D

To verify the effectiveness of the I3D dual modality branch, we carried out experiments using only the RGB branch, only the optical flow branch, and using both paths. The results in Table 4 show that the optical flow branch is more effective than the RGB branch. This is because optical flow can effectively extract motion information in the video, representing the athlete's action state, while the RGB branch can only reflect the temporal and spatial location information of the athlete.

**Table 4:** Ablation experiment results of the I3D module on the UNLV-Dive dataset.

Method	RGB	Flow	SRC
1	×	√	0.8811
2	√	×	0.8720
3 (Complete)	√	√	0.9072

#### 4.4.3 Effect of key frames

As described in Section 3.3.1, before performing the simulation, it is necessary to use yolov5 to extract the key frames of the human body. Different key frames will affect the modeling of water entry posture and splash state, affecting the generation of local features, and thus affecting the final result prediction. Therefore, it is necessary to analyze the key frames separately and observe their impact on the final results. Since this key frame is an innovative concept proposed by us, there is no ground truth in the dataset itself, and it is difficult to directly obtain the accuracy of key frame prediction, so we used another way of thinking to perform ablation experiments. Specifically, we replaced the key frames with the following three variants. (1) Random frame, randomly selecting a frame from the video as the key frame. (2) The last frame before the frame where yolov5 predicts that the person and water are in contact, as the key frame. (3) The next frame where yolov5 predicts that the person and water are in contact, as the key frame. The results in Table 5 show that the random frame has the worst effect because the information is chaotic and there is no rule to follow. The local features generated on this basis may even contain negative information that is useless for the final prediction. The effect of the last and next frames is significantly reduced compared to the frame predicted by yolov5 where the person and water are just in contact. This indicates that this frame contains rich fine-grained object information, which can fully characterize the splash state after entering the water. In addition, it is worth noting that the last frame is better than the next frame, because there is no splash in the last frame, which is more similar to the key frame predicted by yolov5 in our method, while in the next frame, the person has already fallen into the water. The picture contains splash information, and it is extremely difficult to model the scene when the water is just entered.

**Table 5:** Ablation experiment results of the key frames on the UNLV-Dive dataset.

Key Frame	SRC
Random	0.8523
Last	0.8926
Next	0.8798
Ours	0.9072

#### 4.4.4 The role of the FEA module

Finally, we also conducted an ablation analysis of our innovative FEA local feature extraction module. We have used different methods to extract local features for comparative experiments. They are (1) randomly generating the state of the splash, that is, the height and radius. (2) Manually measure the size of the largest splash in the video, and then map it to the scale of the real scene to obtain splash shape information. (3) Crop the largest splash area of the video and use Resnet to extract the splash feature, representing the splash state. The results in Table 6 show that compared with other methods, our FEA module extracts local features the best. This is because the finite element mechanical model based on the CEL algorithm can realistically simulate the change in the water surface after the athlete enters the water (splash shape). Its form is three-dimensional, compared to the two-dimensional video, the depth information enriches the dimensional state of the splash, and the entire process reproduces the entire process of the athlete entering the water, and the generated splash form is true and reliable.

**Table 6:** Ablation experiment results of the FEA module on the UNLV-Dive dataset.

Method	SRC
Random	0.8551
Manual	0.8784
Resnet	0.8909
Ours (FEA)	0.9072

## 5 Conclusions

This study proposes a novel Multi-granularity Extraction Approach for Temporal-spatial features in judge scoring prediction (MEAT) for diving quality assessment, which closely follows the rules of Olympic diving competitions, and re-examines the spatio-temporal feature modeling method of videos in the action quality assessment (AQA) system. On the one hand, dual-modal I3D (RGB and Flow) is used to extract the spatio-temporal features of the diving video granularity and fuse them to form a global feature. On the other hand, human body pose modeling is used to solve the athlete's three-dimensional splash state as a local feature on the granularity of the object. Concatenating the global and local features and based on the probability distribution of the mean value of the referee's score output by label distribution learning (LDL), the score with the highest probability is selected and multiplied by the difficulty coefficient to obtain the diving score. In the future, we plan to apply this new method of multigrain fusion to other sports events in addition to diving. In addition, it is also an interesting direction to explore the interpretability of AQA models (for example, understanding how the network obtains the score for a specific action), which is very important for real-world applications.

## Ethics

Not applicable.

## Availability of data

Data generated and utilized for analyses of results presented in this manuscript are available from the corresponding author on reasonable requests.

## Data availability statement

Data will be made available on request.

## Additional information

No additional information is available for this paper.

## CRediT authorship contribution statement

**Ning Hao:** Investigation, Writing – original draft. **Sihan Ruan:** Conceptualization. **Yiheng Song:** Writing – review & editing. **Jiashun Chen:** Software. **Longgang Tian:** Formal analysis, Validation.

## Declaration of competing interest

The authors declare the following financial interests/personal relationships which may be considered as potential competing interests: Yiheng Song has a patent 'A fine-grained engineering mechanics diving action simulation and evaluation method' licensed to CN113343774 A.
